# Relationship between hyperlipidemia and the risk of death in aneurysm: a cohort study on patients of different ages, genders, and aneurysm locations

**DOI:** 10.3389/fphys.2023.1081395

**Published:** 2023-06-20

**Authors:** Dianzhu Ding, Yongbin Yang, Guangwei Jiang, Yanhui Peng

**Affiliations:** ^1^ Department of Surgery, Hebei Medical University, Shijiazhuang, China; ^2^ Department of Surgery, Hebei General Hospital, Shijiazhuang, China; ^3^ Department of Hepatobiliary Surgery, Hebei General Hospital, Shijiazhuang, China

**Keywords:** hyperlipidemia, aneurysm death, age, gender, aneurysm location

## Abstract

**Aims:** The study aimed to assess the association of hyperlipidemia and the risk of death in the aneurysm population, focusing on age, gender, and aneurysm location differences.

**Methods:** All patients’ data on this retrospective cohort study were obtained from the Medical Information Mart for Intensive Care (MIMIC-III) database, and the baseline characteristics and laboratory parameters of all patients were collected. The COX regression model was established to explore the association of hyperlipidemia and the risk of death for patients with aneurysms. More importantly, subgroup analyses based on the age, gender, and aneurysm location differences were performed.

**Results:** A total of 1,645 eligible patients were enrolled in this study. These patients were divided into the survival group (*n* = 1,098) and the death group (*n* = 547), with a total mortality rate of approximately 33.25%. The result displayed that hyperlipidemia was associated with a decreased death risk in aneurysm patients. In addition, we also found that hyperlipidemia was associated with a lower death risk of abdominal aortic aneurysm and thoracic aortic arch aneurysm among aneurysm patients aged ≥60 years; hyperlipidemia was only a protective factor for the death risk of male patients diagnosed with abdominal aortic aneurysm. For female patients diagnosed with abdominal aortic aneurysm and thoracic aortic arch aneurysm, hyperlipidemia was associated with a decreased death risk.

**Conclusion:** The relationship of hyperlipidemia, hypercholesterolemia, and the risk of death for patients diagnosed with aneurysms was significantly associated with age, gender, and aneurysm location.

## Introduction

An aneurysm is a persistent dilation of the vascular wall caused by a lesion or damage of an artery wall, which is commonly an asymptomatic disease but could cause death because of artery ruptures (Schmitz-Rixen et al., 2016; Liu et al., 2020). It is estimated that there are around 200,000 aneurysm-related deaths worldwide each year (Liu et al., 2020). Nowadays, several literature reports have proposed that aneurysm is one of the typical gender-related aorta diseases (Sweeting et al., 2012; Deery et al., 2017; Boese et al., 2018). In the study by Boese and colleagues, they pointed out that abdominal aortic aneurysms were more likely to occur in men, but women were at a greater risk of rupture and had a worse prognosis; moreover, women who were diagnosed with abdominal aortic aneurysms commonly were older than men (Boese et al., 2018). Understanding gender and age differences of aneurysm would help make more accurate prognosis methods for patients.

Hyperlipidemia is a pathological condition, in which the lipid concentration exceeds normal levels due to the disorder of lipid metabolism in the human body (He and Ye, 2020). Several clinical studies have indicated that hyperlipidemia could affect heart function by promoting the development of atherosclerosis and increasing the risk of non-ischemic heart failure and coronary heart disease, which has been proven to be associated with an increased risk of cardiovascular disease (El-Tantawy and Temraz, 2019; Yao et al., 2020). Nevertheless, to the best of our knowledge, a number of studies have pointed out that hyperlipidemia appears to have a protective effect on the death of patients with aneurysms. There were few studies that comprehensively evaluated the relationship between hyperlipidemia and death due to aneurysms among patients of different ages and genders so far (Cheng et al., 2019; Huber et al., 2019). Understanding the effect of hyperlipidemia on death in aneurysm patients of different ages or genders plays an important role in improving the prognosis of different populations.

Herein, the aims of the present study are to investigate the association of hyperlipidemia and death in the population with aneurysms, focusing on age, gender, and aneurysm location differences.

## Methods

### Data sources and study design

All data on this retrospective cohort study were obtained from the Medical Information Mart for Intensive Care (MIMIC-III) database (version 1.4), which is a large, single-center, freely available database and contains information related to 53,423 patients admitted to the intensive care unit of a large tertiary hospital between 2001 and 2012 (Johnson et al., 2016; Long et al., 2021).

A total of 1,793 patients diagnosed with aneurysms were extracted from the MIMIC-III database. We excluded some patients who had ruptured aneurysms or had abnormal age records. Due to the public availability of the MIMIC-III database, all patients’ private information has been anonymized and does not require approval from the local ethics committee.

### Data collection

We collected the baseline characteristics and laboratory parameters of all patients (Cheng et al., 2019; Jeon-Slaughter et al., 2019), including gender, age, the history of diseases [chronic obstructive pulmonary disease (COPD), lung cancer, atrial fibrillation (AF), liver cirrhosis, congestive heart failure, heart disease, diabetes mellitus, respiratory failure, hyperlipidemia, renal failure, cancer, hypertension, and hypercholesterolemia], respiratory rate, temperature, heart rate, systolic blood pressure (SBP), diastolic blood pressure (DBP), mean arterial pressure (MAP), oxygen saturation (SpO2 %), white blood cell count (WBC, 10^3^/uL), red blood cell count (RBC, 10^3^/uL), sodium (mEq/L), potassium (mEq/L), calcium (mg/dL), platelets (PLT, k/uL), international normalized ratio (INR), mean corpuscular volume (MCV, 10 g/L), glucose (mg/dL), creatinine (mg/dL), blood urea nitrogen (BUN, mg/dL), bicarbonate (mEq/L), hematocrit (%), mean corpuscular hemoglobin concentration (MCHC, %), red cell distribution width (RDW, %), simplified acute physiology score II (SAPSII), sequential organ failure assessment (Sofa), and the history of medication use, such as atorvastatin, imipenem–cilastatin, lovastatin, nystatin, pravastatin, simvastatin, statins, and other lipid-lowering drugs (ezetimibe, cholestyramine, colestipol, colesevelam, ciprofibrate, fenofibrate, gemfibrozil, omega-3, and niacin).

### Outcomes

The primary outcome of our study was death. Hyperlipidemia was diagnosed in terms of the Chinese guidelines for the management of dyslipidemia in adults (Chen et al., 2020); hypercholesterolemia was defined as the condition where patients were treated with antihyperlipidemic agents or had a total cholesterol level ≥220 mg/dL (Kang et al., 2017). Aneurysms included abdominal aortic aneurysms, thoracic aortic arch aneurysms, cerebral aneurysms, and others [including aneurysm of the artery of the lower extremity, aneurysm of the artery of the neck, aneurysm of the artery of the upper extremity, aneurysm of coronary vessels, aneurysm of the heart (wall), aneurysm of the iliac artery, aneurysm of other specified arteries, aneurysm of other visceral arteries, aneurysm of the pulmonary artery, aneurysm of the renal artery, aneurysm of the subclavian artery, aortic aneurysm of unspecified sites without mention of rupture, other aneurysms of the heart, and thoracoabdominal aneurysm without mention of rupture] in the study. The starting date of follow-up was the date of the patient’s admission. All patients were followed for 10 years.

### Statistical analysis

The measurement data on the normal distribution were described by mean ± standard deviation (mean ± SD), and comparison between groups was performed by an independent sample *t*-test. The measurement data on the non-normal distribution used the median and quartile spacing [M (Q1, Q3)], and the Mann–Whitney U rank-sum test was adopted to compare the two groups. The categorical data were conducted by the number of cases and composition ratio n (%), and were compared by the chi-squared or Fisher’s exact test. Several variables with many missing data were deleted (more than 10%). We interpolated the missing data by using R mice, and the sensitivity analysis of data after interpolation is shown in Supplementary Table S1.

First, we conducted a descriptive statistical analysis by univariate difference analysis. Then, univariate COX analysis was carried out to explore the confounding factors. Next, a COX regression model was established with hyperlipidemia and hypercholesterolemia as independent variables and death within 10 years as the dependent variable. Model 1 was regarded as unadjusted. Model 2 adjusted the age, COPD, lung cancer, AF, liver cirrhosis, congestive heart failure, heart disease, diabetes mellitus, respiratory failure, and cancer. Model 3 adjusted several covariates that included age, respiratory rate, heart rate, DBP, MAP, SpO2, WBC, sodium, potassium, PLT, INR, MCV, glucose, creatinine, BUN, MCHC, RDW, SAPSII, Sofa, imipenem–cilastatin, COPD, lung cancer, AF, liver cirrhosis, congestive heart failure, heart disease, diabetes mellitus, respiratory failure, and cancer. More importantly, we assessed the relationships between hyperlipidemia, hypercholesterolemia, and death in patients with aneurysms based on the age, gender, and type of aneurysms. We adopted restricted cubic spline (RCS) curves to assess the dose-response relationship between age and death risk. The hazard ratio (HR) and 95% confidence interval (CI) were calculated in the study. *p* < 0.05 was considered statistically significant.

## Results

### Baseline characteristics

After excluding patients who had ruptured aneurysms (*n* = 87) and had abnormal age records (*n* = 61), a total of 1,645 eligible patients were enrolled in this study, with an average age of 67.17 ± 14.20 years old. These patients were divided into the survival group (*n* = 1,098) and the death group (*n* = 547), with a total mortality rate of approximately 33.25%. There were 968 men and 677 women, and the incidence of male and female death was approximately 32.64% and 34.12%, respectively. Detailed baseline data on all eligible patients are shown in Table 1.

**TABLE 1:** Baseline characteristics of all included participants.

Variable	Total (*n* = 1,645)	Survival group (*n* = 1,098)	Death group (*n* = 547)
Gender, n (%)			
Male	968 (58.84)	652 (59.38)	316 (57.77)
Female	677 (41.16)	446 (40.62)	231 (42.23)
Age, mean ± SD	67.17 ± 14.20	63.75 ± 14.16	74.04 ± 11.54
COPD, n (%)			
No	1,381 (83.95)	978 (89.07)	403 (73.67)
Yes	264 (16.05)	120 (10.93)	144 (26.33)
Lung cancer, n (%)			
No	1,638 (99.57)	1,097 (99.91)	541 (98.90)
Yes	7 (0.43)	1 (0.09)	6 (1.10)
AF, n (%)			
No	1,061 (64.50)	778 (70.86)	283 (51.74)
Yes	584 (35.50)	320 (29.14)	264 (48.26)
Liver cirrhosis, n (%)			
No	1,595 (96.96)	1,074 (97.81)	521 (95.25)
Yes	50 (3.04)	24 (2.19)	26 (4.75)
Congestive heart failure, n (%)			
No	1,207 (73.37)	902 (82.15)	305 (55.76)
Yes	438 (26.63)	196 (17.85)	242 (44.24)
Heart disease, n (%)			
No	1,473 (89.54)	1,012 (92.17)	461 (84.28)
Yes	172 (10.46)	86 (7.83)	86 (15.72)
Diabetes mellitus, n (%)			
No	1,380 (83.89)	953 (86.79)	427 (78.06)
Yes	265 (16.11)	145 (13.21)	120 (21.94)
Respiratory failure, n (%)			
No	1,384 (84.13)	1,001 (91.17)	383 (70.02)
Yes	261 (15.87)	97 (8.83)	164 (29.98)
Hyperlipidemia, n (%)			
No	978 (59.45)	613 (55.83)	365 (66.73)
Yes	667 (40.55)	485 (44.17)	182 (33.27)
Renal failure, n (%)			
No	1,271 (77.26)	950 (86.52)	321 (58.68)
Yes	374 (22.74)	148 (13.48)	226 (41.32)
Cancer, n (%)			
No	1,364 (82.92)	953 (86.79)	411 (75.14)
Yes	281 (17.08)	145 (13.21)	136 (24.86)
Respiratory rate, mean ± SD	16.37 ± 5.16	15.57 ± 4.68	17.98 ± 5.70
Temperature, °C, mean ± SD	36.21 ± 2.16	36.15 ± 2.06	36.32 ± 2.33
Heart rate, mean ± SD	81.19 ± 16.43	79.49 ± 15.02	84.60 ± 18.50
SBP, mean ± SD	126.22 ± 24.30	125.61 ± 22.55	127.43 ± 27.46
DBP, mean ± SD	64.48 ± 14.97	65.14 ± 14.28	63.17 ± 16.18
MAP, mean ± SD	83.92 ± 17.16	84.56 ± 16.19	82.63 ± 18.91
SpO2, %, mean ± SD	97.80 ± 3.88	98.19 ± 3.41	97.02 ± 4.58
WBC (10^3^/uL), M (Q_1_, Q_3_)	9.30 (7.10, 12.50)	9.20 (6.90, 12.30)	9.80 (7.30, 13.40)
RBC (10^3^/uL), mean ± SD	3.82 ± 0.75	3.82 ± 0.76	3.81 ± 0.74
Sodium (mEq/L), mean ± SD	139.18 ± 3.71	139.48 ± 3.51	138.59 ± 4.03
Potassium (mEq/L), mean ± SD	4.21 ± 0.67	4.16 ± 0.58	4.32 ± 0.82
Calcium (mg/dL), mean ± SD	8.53 ± 0.77	8.52 ± 0.72	8.54 ± 0.86
PLT (k/uL), M (Q_1_, Q_3_)	206.00 (153.00, 266.00)	203.00 (151.00, 262.00)	218.00 (156.00, 278.00)
INR, M (Q_1_, Q_3_)	1.20 (1.10, 1.40)	1.20 (1.10, 1.40)	1.20 (1.10, 1.40)
MCV, 10 g/L, mean ± SD	89.57 ± 6.46	89.02 ± 5.91	90.68 ± 7.32
Glucose (mg/dL), M (Q_1_, Q_3_)	121.00 (102.00, 146.00)	120.00 (102.00, 142.00)	123.00 (103.00, 157.00)
Creatinine (mg/dL), M (Q_1_, Q_3_)	0.90 (0.70, 1.20)	0.90 (0.70, 1.10)	1.10 (0.80, 1.60)
BUN (mg/dL), M (Q_1_, Q_3_)	17.00 (13.00, 24.00)	16.00 (12.00, 21.00)	22.00 (16.00, 34.00)
Bicarbonate (mEq/L), mean ± SD	24.78 ± 3.83	24.74 ± 3.34	24.86 ± 4.67
Hematocrit, %, mean ± SD	34.06 ± 6.51	33.92 ± 6.62	34.33 ± 6.26
MCHC, %, mean ± SD	34.04 ± 1.43	34.24 ± 1.34	33.63 ± 1.51
RDW, %, mean ± SD	14.39 ± 1.74	14.04 ± 1.40	15.08 ± 2.11
SAPSII, M (Q_1_, Q_3_)	32.00 (24.00, 40.00)	29.00 (21.00, 37.00)	37.00 (30.00, 44.00)
Sofa, M (Q_1_, Q_3_)	4.00 (2.00, 8.00)	4.00 (2.00, 7.00)	5.00 (3.00, 8.00)
Hypertension, n (%)			
No	575 (34.95)	387 (35.25)	188 (34.37)
Yes	1,070 (65.05)	711 (64.75)	359 (65.63)
Hypercholesterolemia, n (%)			
No	1,391 (84.56)	912 (83.06)	479 (87.57)
Yes	254 (15.44)	186 (16.94)	68 (12.43)
Atorvastatin, n (%)			
No	1,157 (70.33)	785 (71.49)	372 (68.01)
Yes	488 (29.67)	313 (28.51)	175 (31.99)
Imipenem–cilastatin, n (%)			
No	1,638 (99.57)	1,096 (99.82)	542 (99.09)
Yes	7 (0.43)	2 (0.18)	5 (0.91)
Lovastatin, n (%)			
No	1,642 (99.82)	1,096 (99.82)	546 (99.82)
Yes	3 (0.18)	2 (0.18)	1 (0.18)
Nystatin, n (%)			
No	1,642 (99.82)	1,097 (99.91)	545 (99.63)
Yes	3 (0.18)	1 (0.09)	2 (0.37)
Pravastatin, n (%)			
No	1,593 (96.84)	1,064 (96.90)	529 (96.71)
Yes	52 (3.16)	34 (3.10)	18 (3.29)
Simvastatin, n (%)			
No	1,320 (80.24)	870 (79.23)	450 (82.27)
Yes	325 (19.76)	228 (20.77)	97 (17.73)
Statins, n (%)			
No	817 (49.67)	551 (50.18)	266 (48.63)
Yes	828 (50.33)	547 (49.82)	281 (51.37)
Other lipid-lowering drugs, n (%)			
No	1,594 (96.90)	1,061 (96.63)	533 (97.44)
Yes	51 (3.10)	37 (3.37)	14 (2.56)

Other lipid-lowering drugs: ezetimibe, cholestyramine, colestipol, colesevelam, ciprofibrate, fenofibrate, gemfibrozil, omega-3, and niacin; COPD, chronic obstructive pulmonary disease; AF, atrial fibrillation; SBP, systolic blood pressure; DBP, diastolic blood pressure; MAP, mean arterial pressure; SpO2, oxygen saturation; WBC, white blood cell count; RBC, red blood cell count; PLT, platelet; INR, international normalized ratio; MCV, mean corpuscular volume; BUN, blood urea nitrogen; MCHC, mean corpuscular hemoglobin concentration; RDW, red cell distribution width; SAPSII, simplified acute physiology score II; Sofa, sequential organ failure assessment.

### Assessment of confounding factors by univariate Cox regression analysis

We performed a univariate Cox regression to analyze the possible confounding factors related to death in patients diagnosed with aneurysms. Supplementary Table S2 indicates that age, COPD, lung cancer, AF, liver cirrhosis, congestive heart failure, heart disease, diabetes mellitus, respiratory failure, renal failure, cancer, respiratory rate, heart rate, DBP, MAP, SpO2, WBC, sodium, potassium, PLT, INR, MCV, glucose, creatinine, BUN, MCHC, RDW, SAPSII, Sofa, and imipenem–cilastatin might be associated with the risk of death for patients with aneurysms (*p* < 0.05).

### The relationship between hyperlipidemia, hypercholesterolemia, and death risk of aneurysm patients

The effects of hyperlipidemia on the death risk of aneurysm patients are presented in Table 2. Model 1 displayed that hyperlipidemia could decrease the death risk of aneurysm patients (HR = 0.69, 95% CI: 0.58–0.83, *p* < 0.001), with a similar result in models 2 (HR = 0.51, 95% CI: 0.42–0.61, *p* < 0.001) and Model 3 (HR = 0.52, 95% CI: 0.43–0.62, *p* < 0.001). In addition, we found that hypercholesterolemia was also associated a reduced risk of aneurysm patients’ death in Model 1 and Model 2. Nevertheless, after adjusting covariates, Model 3 demonstrated that there was no statistically significant difference between hypercholesterolemia and death (HR = 0.77, 95% CI: 0.60–1.01, *p* = 0.055).

**TABLE 2:** Relationship between hyperlipidemia, hypercholesterolemia, and the death risk of aneurysm patients.

Variables	Sample size	Model 1	*P*	Model 2	*P*	Model 3	*P*
		HR (95%CI)		HR (95%CI)		HR (95%CI)	
Non-hyperlipidemia	978	Reference		Reference		Reference	
Hyperlipidemia	667	0.69 (0.58–0.83)	<0.001	0.51 (0.42–0.61)	<0.001	0.52 (0.43–0.62)	<0.001
Non-hypercholesterolemia	1,391	Reference		Reference		Reference	
Hypercholesterolemia	254	0.73 (0.57–0.94)	0.016	0.69 (0.53–0.88)	0.004	0.77 (0.60–1.01)	0.055

HR: hazard ratio; CI: confidence interval.

Model 1: unadjusted.

Model 2: adjusted age, chronic obstructive pulmonary disease (COPD), lung cancer, atrial fibrillation (AF), liver cirrhosis, congestive heart failure, heart disease, diabetes mellitus, respiratory failure, and cancer.

Model 3: adjusted age, respiratory rate, heart rate, diastolic blood pressure, mean arterial pressure, oxygen saturation, white blood count, sodium, potassium, platelets, international normalized ratio, mean corpuscular volume, glucose, creatinine, blood urea nitrogen, mean corpuscular hemoglobin concentration, red cell distribution width, simplified acute physiology score II, sequential organ failure assessment, imipenem–cilastatin; COPD, lung cancer; AF, liver cirrhosis, congestive heart failure, heart disease, diabetes mellitus, respiratory failure, and cancer.

### Subgroup analysis was performed based on age, gender, and aneurysm location

**FIGURE 1:**
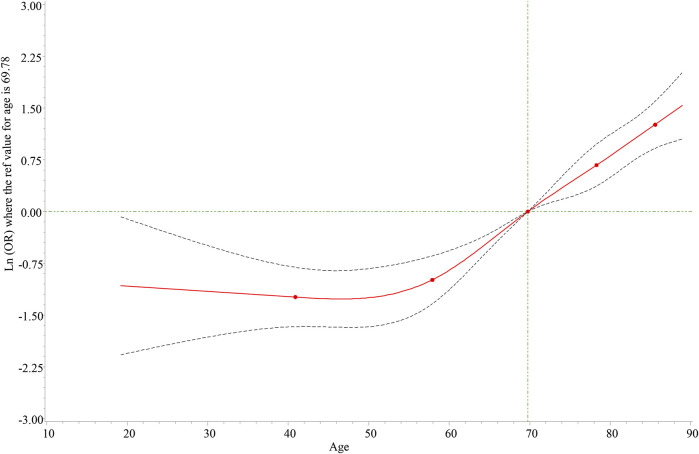
Dose-response relationship between age and death risk. The solid line represents the odds ratios, and the dotted line represents the 95% confidence interval.

We could find that there was a non-linear relationship between age and death in the RCS graph (Figure 1), and the relationship was statistically significant (*p* = 0.001). Interestingly, there was a protective trend for patients aged <60 years and a risk trend for patients aged ≥60 years. Herein, we discussed the relationship between hyperlipidemia, hypercholesterolemia, and the death risk of different aneurysm locations based on patients of different ages. As shown in Table 3, hyperlipidemia was associated with a lower death risk of abdominal aortic aneurysm, thoracic aortic arch aneurysm, and others among aneurysm patients aged ≥60 years. However, there was no statistically significant difference between hyperlipidemia and death of different aneurysm locations in patients aged <60 years. Simultaneously, the association of hypercholesterolemia and the death risk of different aneurysm locations is also analyzed in Table 3. The result indicated that hypercholesterolemia could decrease the death risk of other aneurysm locations in patients aged ≥60 years, while hypercholesterolemia was a risk factor for patients aged <60 years. Similarly, we performed the subgroup analysis based on gender and the aneurysm location. Table 4 implies that hyperlipidemia was only a protective factor for the death risk of male patients diagnosed with abdominal aortic aneurysms. For female patients diagnosed with abdominal aortic aneurysms and thoracic aortic arch aneurysms, hyperlipidemia was still associated with a decreased death risk.

**TABLE 3:** Subgroup analysis was performed based on the age and aneurysm location.

Aneurysm locations	Variable	Sample size	<60 years	*P*	Sample size	≥60 years	*P*
			HR (95% CI)			HR (95% CI)	
Abdominal aortic aneurysm	Non-hyperlipidemia	40	Reference		471	Reference	
	Hyperlipidemia		N/A			0.48 (0.36–0.65)	<0.001
	Non-hypercholesterolemia		Reference			Reference	
	Hypercholesterolemia		N/A			0.75 (0.49–1.15)	0.191
Thoracic aortic arch aneurysm	Non-hyperlipidemia	151	Reference		280	Reference	
	Hyperlipidemia		7.95 (0.91–69.51)	0.061		0.57 (0.33–0.98)	0.042
	Non-hypercholesterolemia		Reference			Reference	
	Hypercholesterolemia		0.83 (0.06–12.63)	0.894		0.82 (0.41–1.66)	0.583
Cerebral aneurysm	Non-hyperlipidemia	172	Reference		142	Reference	
	Hyperlipidemia		0.81 (0.15–4.38)	0.802		0.33 (0.11–1.02)	0.054
	Non-hypercholesterolemia		Reference			Reference	
	Hypercholesterolemia		3.94 (0.30–52.60)	0.299		0.48 (0.11–2.00)	0.310
Others	Non-hyperlipidemia	104	Reference		285	Reference	
	Hyperlipidemia		2.55 (0.16–41.06)	0.510		0.46 (0.30–0.70)	<0.001
	Non-hypercholesterolemia		Reference			Reference	
	Hypercholesterolemia		632.62 (9.73–41126.69)	0.003		0.55 (0.31–0.96)	0.036

HR: hazard ratio; CI: confidence interval; NA: the sample size was insufficient to fit.

Others: include aneurysm of the artery of the lower extremity, aneurysm of the artery of the neck, aneurysm of the artery of the upper extremity, aneurysm of coronary vessels, aneurysm of the heart (wall), aneurysm of the iliac artery, aneurysm of other specified arteries, aneurysm of other visceral arteries, aneurysm of the pulmonary artery, aneurysm of the renal artery, aneurysm of the subclavian artery, aortic aneurysm of the unspecified site without a mention of rupture, other aneurysm of the heart, and thoracoabdominal aneurysm without a mention of rupture.

**TABLE 4:** Subgroup analysis was performed based on the gender and aneurysm location.

Aneurysm location	Variable	Sample size	Male	*P*	Sample size	Female	*P*
			HR (95%CI)			HR (95%CI)	
Abdominal aortic aneurysm	Non-hyperlipidemia	358	Reference		153	Reference	
	Hyperlipidemia		0.40 (0.27–0.58)	<0.001		0.48 (0.27–0.87)	0.015
	Non-hypercholesterolemia		Reference			Reference	
	Hypercholesterolemia		0.83 (0.58–1.44)	0.504		0.78 (0.36–1.70)	0.535
Thoracic aortic arch aneurysm	Non-hyperlipidemia	281	Reference		150	Reference	
	Hyperlipidemia		1.18 (0.61–2.25)	0.626		0.19 (0.06–0.61)	0.006
	Non-hypercholesterolemia		Reference			Reference	
	Hypercholesterolemia		1.49 (0.67–3.33)	0.331		0.35 (0.07–1.83)	0.214
Cerebral aneurysm	Non-hyperlipidemia	99	Reference		215	Reference	
	Hyperlipidemia		0.23 (0.02–3.11)	0.266		0.63 (0.23–1.78)	0.385
	Non-hypercholesterolemia		Reference			Reference	
	Hypercholesterolemia		0.51 (0.03–7.89)	0.629		0.64 (0.18–2.27)	0.485
Others	Non-hyperlipidemia	230	Reference		159	Reference	
	Hyperlipidemia		0.65 (0.38–1.12)	0.121		0.40 (0.22–0.76)	0.005
	Non-hypercholesterolemia		Reference			Reference	
	Hypercholesterolemia		0.91 (0.48–1.75)	0.787		0.50 (0.18–1.36)	0.174

HR: hazard ratio; CI: confidence interval; Others: include aneurysm of the artery of the lower extremity, aneurysm of the artery of the neck, aneurysm of the artery of the upper extremity, aneurysm of coronary vessels, aneurysm of the heart (wall), aneurysm of the iliac artery, aneurysm of other specified arteries, aneurysm of other visceral arteries, aneurysm of the pulmonary artery, aneurysm of the renal artery, aneurysm of the subclavian artery, aortic aneurysm of the unspecified site without a mention of rupture, other aneurysm of the heart, and thoracoabdominal aneurysm without a mention of rupture.

## Discussion

Aneurysms are generally asymptomatic and are not diagnosed until a serious complication occurs, such as aortic rupture or dissection (Pinard et al., 2019). It is reported that there were nearly 9,000 deaths annually caused by aneurysms, and ruptured abdominal aortic aneurysms have been the 13th leading cause of death in the United States, which posed a health crisis and economic burden for most families (Sen et al., 2021). Nowadays, deaths from aneurysms have attracted widespread attention. Several studies have also proposed that these deaths can be prevented if the patients at risk could be identified and through appropriate aneurysm management (Yiu and Cheng, 2016; Algra et al., 2019). In this retrospective cohort study, we reported the relationships between hyperlipidemia, hypercholesterolemia, and death risk in patients diagnosed with aneurysms, and expounded that hyperlipidemia could decrease the death risk. More importantly, we explored the association of hyperlipidemia, hypercholesterolemia, and death based on the aneurysm location, age, and gender differences.

Our study stated that hyperlipidemia was a protective factor against death among patients diagnosed with aneurysms, which was supported by previous research studies (Cheng et al., 2019; Huber et al., 2019). A meta-analysis also suggested that hyperlipidemia may significantly decrease the risk of cerebral aneurysm rupture, and the benefits appear to be independent of statin therapy (Cheng et al., 2019). This may be attributed to the direct vascular effects of hyperlipidemia on the aortic wall, thereby impeding the progression of an aneurysm (Zhang et al., 2015; Huang et al., 2018). In addition, some studies also showed that hyperlipidemia’s protective effect may be related to statin therapy (Salata et al., 2018; Xiong et al., 2022). However, the specific protective mechanism remains unclear and needs to be further explored. In the study by Huang, et al., they found that males were associated with a higher risk in the onset and progression of abdominal aortic aneurysm, and men had a higher risk of death from rupture and vasodilation than women after surgery (Huang et al., 2016). In addition, Mahaney and co-workers also found that the morbidity and mortality of aneurysm patients during surgery and hospitalization increased with age (Mahaney et al., 2014). These findings suggest that gender and age differences need to be considered when investigating the relation of hyperlipidemia and hypercholesterolemia, and the risk of death in patients with aneurysms. Interestingly, in the present study, we found different relationships between hyperlipidemia, hypercholesterolemia, and death based on the aneurysm location, age, and gender. To the best of our knowledge, no relevant research has reported this result to date.

Specifically speaking, our study showed that hyperlipidemia was advantageous for the prognosis for patients with abdominal aortic aneurysms aged ≥60 years or female patients with thoracic aortic arch aneurysms aged ≥60 years. Although most studies have reported a favorable effect of hyperlipidemia on the prognosis of aneurysm patients, this study further demonstrated that the relationship between hyperlipidemia and the risk of death for patients diagnosed with aneurysms was significantly associated with age, gender, and aneurysm location. Noteworthily, in terms of the relationship between hypercholesterolemia and risk of death for patients with aneurysms, we found that there was no statistically significant difference, and the reason might be associated with the relatively small sample size. Although the aforementioned guesses may support our findings, we still lack direct experimental evidence. Our conclusion needs to be confirmed by more related studies.

As far as we know, this was the first detailed cohort study regarding the association between hyperlipidemia, hypercholesterolemia, and death based on the aneurysm location, age, and gender. We believed the findings could provide an early warning for clinicians to consider the difference in age, gender, and aneurysm location in assessing the death risk of aneurysms. However, our study was inevitably linked with some limitations. First, there was a relatively small sample size in the study, which might have limited the statistical power, but our study has a long enough follow-up period in investigating the association. Second, due to all data being derived from the MIMIC-III database, we did not collect data related to the surgical treatment, which might also be responsible for the different associations between hyperlipidemia, hypercholesterolemia, and the risk of death in aneurysm populations (Huber et al., 2019). Third, due to the retrospective nature of this study, potential subjective biases may occur during data collection. More studies are needed to explore the association.

## Conclusion

In conclusion, our study indicated that the relationship between hyperlipidemia, hypercholesterolemia, and the risk of death for patients diagnosed with aneurysms was significantly associated with age, gender, and aneurysm location, which implied that future research practices and guidelines need to consider the difference in age, gender, and aneurysm location in assessing and treating aneurysms.

## Data Availability

Publicly available datasets were analyzed in this study. These data can be found in the MIMIC-III database, https://mimic.physionet.org/iii/.
