# Supplementary material for: Relationship between hyperlipidemia and the risk of death in aneurysm: a cohort study on patients of different ages, genders, and aneurysm locations
Source: Front Physiol. 2023 Jun 20;14:1081395. doi: 10.3389/fphys.2023.1081395 (PMC10318894; doi:10.3389/fphys.2023.1081395)
Supplement: Supplementary file 1 [file Table1.DOCX]

**Supplemental Table 1** The sensitivity analysis of missing data before and after interpolation

| Variables | Miss, n (%) | Before the interpolation | After the interpolation | Statistics | *P* |
| --- | --- | --- | --- | --- | --- |
| Age, Mean ± SD | 39 (2.37) | 67.17 ± 14.20 | 67.31 ± 14.27 | t=-0.28 | 0.779 |
| Respiratory rate, Mean ± SD | 59 (3.59) | 16.37 ± 5.16 | 16.38 ± 5.24 | t=-0.05 | 0.964 |
| Temperature, ℃, Mean ± SD | 67 (4.07) | 36.21 ± 2.16 | 36.21 ± 2.19 | t=0.02 | 0.984 |
| Heart rate, Mean ± SD | 59 (3.59) | 81.19 ± 16.43 | 81.24 ± 16.64 | t=-0.08 | 0.936 |
| SBP, Mean ± SD | 59 (3.59) | 126.22 ± 24.30 | 126.08 ± 24.64 | t=0.16 | 0.876 |
| DBP, Mean ± SD | 59 (3.59) | 64.48V± 14.97 | 64.44 ± 15.18 | t=0.08 | 0.937 |
| MAP, Mean ± SD | 59 (3.59) | 83.92 ± 17.16 | 83.84 ± 17.41 | t=0.13 | 0.894 |
| SpO2, %, Mean ± SD | 59 (3.59) | 97.80 ± 3.88 | 97.79 ± 3.94 | t=0.10 | 0.921 |
| WBC (10^3^/uL), M (Q_1_, Q_3_) | 16 (0.97) | 9.30 (7.10, 12.50) | 9.30 (7.00, 12.60) | Z=-0.015 | 0.988 |
| RBC (10^3^/uL), Mean ± SD | 16 (0.97) | 3.82 ± 0.75 | 3.82 ± 0.76 | t=-0.00 | 0.999 |
| Sodium (mEq/L), Mean ± SD | 18 (1.09) | 139.18 ± 3.71 | 139.18 ± 3.73 | t=0.03 | 0.980 |
| Potassium (mEq/L), Mean ± SD | 17 (1.03) | 4.21 ± 0.67 | 4.21 ± 0.67 | t=-0.05 | 0.958 |
| Calcium (mg/dL), Mean ± SD | 123 (7.48) | 8.53 ± 0.77 | 8.53 ± 0.79 | t=-0.20 | 0.839 |
| PLT (k/uL), M (Q_1_, Q_3_) | 16 (0.97) | 206.00 (153.00, 266.00) | 206.00 (152.00, 268.00) | Z=0.021 | 0.983 |
| INR, M (Q_1_, Q_3_) | 48 (2.92) | 1.20 (1.10, 1.40) | 1.20 (1.10, 1.40) | Z=-0.192 | 0.848 |
| MCV, Mean ± SD | 16 (0.97) | 89.57 ± 6.46 | 89.57 ± 6.49 | t=0.02 | 0.985 |
| magnesium, Mean ± SD | 37 (2.25) | 1.93 ± 0.44 | 1.93 ± 0.44 | t=-0.08 | 0.936 |
| Glucose (mg/dL), M (Q_1_, Q_3_) | 18 (1.09) | 121.00 (102.00, 146.00) | 121.00 (102.00, 146.00) | Z=0.012 | 0.991 |
| Creatinine (mg/dL), M (Q_1_, Q_3_) | 15 (0.91) | 0.90 (0.70, 1.20) | 0.90 (0.70, 1.20) | Z=-0.003 | 0.998 |
| BUN (mg/dL), M (Q_1_, Q_3_) | 15 (0.91) | 17.00 (13.00, 24.00) | 17.00 (13.00, 25.00) | Z=-0.036 | 0.972 |
| Bicarbonate (mEq/L), Mean ± SD | 18 (1.09) | 24.78 ± 3.83 | 24.78 ± 3.85 | t=0.02 | 0.988 |
| Hematocrit, %, Mean ± SD | 16 (0.97) | 34.06 ± 6.51 | 34.06 ± 6.53 | t=0.01 | 0.991 |
| Hemoglobin (g/dL), Mean ± SD | 16 (0.97) | 11.58 ± 2.26 | 11.58 ± 2.27 | t=0.00 | 0.998 |
| MCHC, %, Mean ± SD | 16 (0.97) | 34.04 ± 1.43 | 34.04 ± 1.43 | t=0.00 | 0.996 |
| RDW, %, Mean ± SD | 16 (0.97) | 14.39 ± 1.74 | 14.39 ± 1.74 | t=-0.03 | 0.974 |
| SAPSII, M (Q_1_, Q_3_) | 39 (2.37) | 32.00 (24.00, 40.00) | 32.00 (24.00, 40.00) | Z=0.288 | 0.773 |
| Sofa, M (Q_1_, Q_3_) | 52 (3.16) | 4.00 (2.00, 8.00) | 4.00 (2.00, 8.00) | Z=0.333 | 0.739 |

**Note:** SBP: systolic blood pressure; DBP: diastolic blood pressure; MAP: mean arterial pressure; SpO2: oxygen saturation; WBC: white blood count; RBC: red blood count; PLT: platelets; INR: international normalized ratio; MCV: mean corpuscular volume; BUN: blood urea nitrogen; MCHC: mean corpuscular hemoglobin concentration; RDW: red cell distribution width; SAPSII: simplified acute physiology score II; Sofa: sequential organ failure assessment.

**Supplemental Table 2** Assessment of confounding factors by univariate Cox regression analysis

| Variables | β | S. E | Z | *P* | HR | Lower | Upper |
| --- | --- | --- | --- | --- | --- | --- | --- |
| Gender |  |  |  |  |  |  |  |
| Male |  |  |  |  | Ref |  |  |
| Female | 0.048 | 0.087 | 0.555 | 0.577 | 1.05 | 0.89 | 1.24 |
| Age | 0.056 | 0.004 | 13.827 | <0.001 | 1.06 | 1.05 | 1.07 |
| COPD |  |  |  |  |  |  |  |
| No |  |  |  |  | Ref |  |  |
| Yes | 0.783 | 0.097 | 8.056 | <0.001 | 2.19 | 1.81 | 2.65 |
| Lung cancer |  |  |  |  |  |  |  |
| No |  |  |  |  | Ref |  |  |
| Yes | 1.789 | 0.411 | 4.357 | <0.001 | 5.98 | 2.67 | 13.40 |
| AF |  |  |  |  |  |  |  |
| No |  |  |  |  | Ref |  |  |
| Yes | 0.686 | 0.086 | 8.011 | <0.001 | 1.99 | 1.68 | 2.35 |
| Liver cirrhosis |  |  |  |  |  |  |  |
| No |  |  |  |  | Ref |  |  |
| Yes | 0.612 | 0.201 | 3.043 | 0.002 | 1.84 | 1.24 | 2.73 |
| Congestive heart failure |  |  |  |  |  |  |  |
| No |  |  |  |  | Ref |  |  |
| Yes | 1.008 | 0.086 | 11.68 | <0.001 | 2.74 | 2.31 | 3.25 |
| Heart disease |  |  |  |  |  |  |  |
| No |  |  |  |  | Ref |  |  |
| Yes | 0.598 | 0.118 | 5.09 | <0.001 | 1.82 | 1.44 | 2.29 |
| Diabetes mellitus |  |  |  |  |  |  |  |
| No |  |  |  |  | Ref |  |  |
| Yes | 0.467 | 0.103 | 4.52 | <0.001 | 1.60 | 1.30 | 1.95 |
| Respiratory failure |  |  |  |  |  |  |  |
| No |  |  |  |  | Ref |  |  |
| Yes | 1.221 | 0.094 | 13.029 | <0.001 | 3.39 | 2.82 | 4.07 |
| Renal failure |  |  |  |  |  |  |  |
| No |  |  |  |  | Ref |  |  |
| Yes | 1.182 | 0.087 | -4.053 | <0.001 | 3.26 | 2.75 | 3.87 |
| Cancer |  |  |  |  |  |  |  |
| No |  |  |  |  | Ref |  |  |
| Yes | 0.634 | 0.099 | 6.408 | <0.001 | 1.89 | 1.55 | 2.29 |
| Respiratory rate | 0.075 | 0.007 | 10.326 | <0.001 | 1.08 | 1.06 | 1.09 |
| Temperature | 0.058 | 0.037 | 1.587 | 0.113 | 1.06 | 0.99 | 1.14 |
| Heart rate | 0.016 | 0.002 | 6.599 | <0.001 | 1.02 | 1.01 | 1.02 |
| SBP | 0.003 | 0.002 | 1.396 | 0.163 | 1.01 | 1.01 | 1.01 |
| DBP | -0.007 | 0.003 | -2.39 | 0.017 | 0.99 | 0.99 | 0.99 |
| MAP | -0.006 | 0.003 | -2.281 | 0.023 | 0.99 | 0.99 | 0.99 |
| SpO2 | -0.043 | 0.007 | -6.627 | <0.001 | 0.96 | 0.95 | 0.97 |
| WBC | 0.028 | 0.008 | 3.544 | <0.001 | 1.03 | 1.01 | 1.04 |
| RBC | -0.032 | 0.057 | -0.564 | 0.573 | 0.97 | 0.87 | 1.08 |
| Sodium | -0.055 | 0.011 | -4.955 | <0.001 | 0.95 | 0.93 | 0.97 |
| Potassium | 0.285 | 0.058 | 4.935 | <0.001 | 1.33 | 1.19 | 1.49 |
| Calcium | 0.034 | 0.057 | 0.604 | 0.545 | 1.03 | 0.93 | 1.16 |
| PLT | 0.002 | 0.000 | 4.153 | <0.001 | 1.01 | 1.01 | 1.01 |
| INR | 0.088 | 0.034 | 2.574 | 0.010 | 1.09 | 1.02 | 1.17 |
| MCV | 0.039 | 0.007 | 5.6 | <0.001 | 1.04 | 1.03 | 1.05 |
| Glucose | 0.004 | 0.001 | 5.376 | <0.001 | 1.01 | 1.01 | 1.01 |
| Creatinine | 0.165 | 0.017 | 9.499 | <0.001 | 1.18 | 1.14 | 1.22 |
| BUN | 0.018 | 0.001 | -51.785 | <0.001 | 1.02 | 1.02 | 1.02 |
| Bicarbonate | 0.008 | 0.012 | 0.646 | 0.519 | 1.01 | 0.98 | 1.03 |
| Hematocrit | 0.007 | 0.007 | 1.115 | 0.266 | 1.01 | 0.99 | 1.02 |
| MCHC | -0.259 | 0.028 | -9.146 | <0.001 | 0.77 | 0.73 | 0.82 |
| RDW | 0.249 | 0.019 | 13.356 | <0.001 | 1.28 | 1.24 | 1.33 |
| SAPSII | 0.036 | 0.003 | 13.125 | <0.001 | 1.04 | 1.03 | 1.04 |
| Sofa | 0.036 | 0.012 | 2.985 | 0.003 | 1.04 | 1.01 | 1.06 |
| Hypertension |  |  |  |  |  |  |  |
| No |  |  |  |  | Ref |  |  |
| Yes | 0.041 | 0.090 | 0.464 | 0.646 | 1.04 | 0.87 | 1.24 |
| Atorvastatin |  |  |  |  |  |  |  |
| No |  |  |  |  | Ref |  |  |
| Yes | 0.138 | 0.092 | 1.508 | 0.131 | 1.15 | 0.96 | 1.37 |
| Imipenem cilastatin |  |  |  |  |  |  |  |
| No |  |  |  |  | Ref |  |  |
| Yes | 1.039 | 0.449 | 2.311 | 0.021 | 2.83 | 1.17 | 6.82 |
| Lovastatin |  |  |  |  |  |  |  |
| No |  |  |  |  | Ref |  |  |
| Yes | 0.208 | 1.001 | 0.210 | 0.835 | 1.23 | 0.17 | 8.75 |
| Nystatin |  |  |  |  |  |  |  |
| No |  |  |  |  | Ref |  |  |
| Yes | 0.655 | 0.708 | 0.924 | 0.355 | 1.93 | 0.48 | 7.72 |
| Pravastatin |  |  |  |  |  |  |  |
| No |  |  |  |  | Ref |  |  |
| Yes | 0.046 | 0.240 | 0.187 | 0.848 | 1.05 | 0.65 | 1.67 |
| Simvastatin |  |  |  |  |  |  |  |
| No |  |  |  |  | Ref |  |  |
| Yes | -0.128 | 0.112 | -1.142 | 0.253 | 0.88 | 0.71 | 1.10 |
| Statins |  |  |  |  |  |  |  |
| No |  |  |  |  | Ref |  |  |
| Yes | 0.070 | 0.086 | 0.813 | 0.416 | 1.07 | 0.91 | 1.27 |
| Other lipid-lowering drugs, n (%) |  |  |  |  |  |  |  |
| No |  |  |  |  | Ref |  |  |
| Yes | -0.219 | 0.271 | -0.811 | 0.418 | 0.80 | 0.47 | 1.37 |

**Note:** Other lipid-lowering drugs: ezetimibe, cholestyramine, colestipol, colesevelam, ciprofibrate, fenofibrate, gemfibrozil, omega-3 and niacin; COPD: chronic obstructive pulmonary disease; AF: atrial fibrillation; SBP: systolic blood pressure; DBP: diastolic blood pressure; MAP: mean arterial pressure; SpO2: oxygen saturation; WBC: white blood count; RBC: red blood count; PLT: platelets; INR: international normalized ratio; MCV: mean corpuscular volume; BUN: blood urea nitrogen; MCHC: mean corpuscular hemoglobin concentration; RDW: red cell distribution width; SAPSII: simplified acute physiology score II; Sofa: sequential organ failure assessment; HR: hazard ratios; CI: confidence interval.
